# Stiripentol for the treatment of refractory status epilepticus

**DOI:** 10.1186/s42466-024-00348-x

**Published:** 2024-10-21

**Authors:** Leona Möller, Ole J. Simon, Clara Jünemann, Meike Austermann-Menche, Marc-Philipp Bergmann, Lena Habermehl, Katja Menzler, Lars Timmermann, Adam Strzelczyk, Susanne Knake

**Affiliations:** 1 Department of Neurology, Epilepsy Center Hessen, Philipps University Marburg, Baldingerstr, 35043 Marburg, Germany; 2 Center for Brain Mind and Behavior (CMBB), Philipps University Marburg, Marburg, Germany; 3 Department of Neurology, Epilepsy Center Frankfurt Rhine-Main, Goethe-University Frankfurt, University Hospital Frankfurt, Frankfurt Am Main, Germany

**Keywords:** Epilepsy, Status epilepticus, Stiripentol

## Abstract

**Background:**

Status epilepticus (SE) is one of the most common neurological emergencies and an acutely life-threatening condition characterized by high mortality and morbidity. Despite the well-established staged therapy of status epilepticus, especially stages 1 and 2, more than one third of patients develop (super-) refractory SE. Despite a large variety of potential treatment options for super-refractory SE, there is an unmet clinical need of potential new treatment ideas in this often desperate clinical situation.

A number of studies have demonstrated the safety and efficacy of stiripentol (STP) in patients with Dravet syndrome (DS) and in children with focal epilepsy and generalized epilepsies. Some smaller series and case reports have documented the use of STP in the treatment of status epilepticus in adult patients.

**Methods:**

We retrospectively analyzed all patients who were admitted to the Department of Neurology at Marburg University Hospital between 2013 and 2023 with a diagnosis of (super)-refractory status epilepticus and who received additional treatment of SE with STP. All patients who received STP during the SE were included, regardless of previous medication.

**Results:**

SE ceased in 64% of 25 patients (13 female and 12 male). The mean age was 58.6 ± 21.9 years (mean ± SD). 72% had a structural epilepsy. In 20% of patients, SE was terminated by the administration of STP alone in 32% of cases, while in a further 32% of patients, the simultaneous administration of multiple anti-seizure medications (ASMs) including STP was potentially responsible for the cessation of the SE, with valproic acid (VPA), benzodiazepines and STP, being the most frequently implicated ASMs. In 12% of patients, there was at least a temporary improvement in the electroencephalogram (EEG). Stiripentol had to be discontinued in three cases due to a reduction in vigilance or hypercalcemia.

**Conclusions:**

Stiripentol may represent a promising additional treatment option for refractory and super-refractory status epilepticus. The tolerability of this treatment has already been demonstrated in previous studies, and was also reflected in these data. Further prospective investigation in larger patient populations are necessary to ascertain the efficacy of stiripentol in SE.

**Trial registration:**

NCT06540378, retrospectively registered.

## Background

With an incidence of 10–40 per 100,000 person-years and a 7–33% mortality, status epilepticus (SE) is one of the most common neurological emergencies. Long-term consequences include neurological, cognitive and behavioural disorders and a significant reduction in quality of life.

Several predictors of poor outcome have been described: advanced age, de novo presentation of epilepsy due to SE, reduced vigilance prior to treatment initiation, and seizure semiology [12]. Sculier et al. have also shown that the incidence of recurrent SE in patients with SE ranges from 13 to 37% in adults and children. In their study, predictors of recurrent SE were age < 4 years, female sex, lack of drug response after the first dose, and symptomatic and progressive aetiologies. Despite following well-established treatment guidelines with staged therapy of status epilepticus, some patients develop (super-)refractory SE. Super-refractory status epilepticus (SRSE) is characterized by unresponsiveness to initial anesthetic therapy. According to Tian et al. from 2014, SRSE occurs in about 12% of all cases of SE [14]. Kantanen et al. gen 75 patients with ICU- and anesthesia-treated RSE, corresponding to an annual incidence of 3.0; 21% of the patients were classified as SRSE, with the annual incidence being 0.6/100 000 [9]. In cases of refractory SE, the administration of ASM, whether approved or not, is gredommended by international treatment guidelines. Reports on various therapeutic approaches for the treatment of super-refractory SE exist, but there is no recommendation for a specific therapy.

Stiripentol (STP) was approved in Europe in 2007 as an orphan drug for the treatment of bilateral tonic–clonic seizures (BTCS) in children with Dravet syndrome (DS) in combination with clobazam (CLB) and valproic acid (VPA) (European Medicines [6]).

Several clinical studies have shown the safety and efficacy of STP in patients with DS (Balestrini and Sisodiya 2017) and also in children with focal epilepsy and generalized or myoclonic seizures [11]. Brigo et al. conducted a Cochrane analysis to evaluate the efficacy and tolerability of STP as an add-on treatment for people with drug-resistant focal epilepsy taking anti-seizure medication (ASM) [3, 4]. The long-term efficacy, tolerability and predictors of response to treatment with STP were retrospectively investigated by Balestrini et al. in 2022 in 196 patients with epilepsy and long-term follow-up (range 0.5–232.8 months). Based on the results of this retrospective cohort study, it was suggested that STP is an effective and well-tolerated treatment option not only for DS, but also for other epilepsy syndromes with or without a confirmed genetic aetiology. The duration of response across different aetiologies was influenced by age at initiation of STP treatment [2].

In an earlier study, we retrospectively investigated 22 adult patients with refractory epilepsies treated with STP from March 2007 to July 2020 to evaluate the safety and efficacy of add-on treatment with STP in adult patients with severe pharmacoresistant focal or multifocal epilepsy. After 6 months, 72.7% of patients were still taking STP and 31% of patients were responders, including 13% who were seizure-free. The 12-month retention rate was 54.4%, the response rate was 36.4% and 13.6% of patients were seizure-free after 12 months of FU. Reasons for discontinuation were increased seizure frequency, hyperammonemia and encephalopathy, even occurring unrelated to a coadministration of valproate [8]. In this series, STP was initiated in five patients during super-refractory status.

epilepticus. Four of these patients were mentioned in the present study.

Some series and case reports describe the use of STP in the treatment of status epilepticus in adult patients, none had more than 10 patients included. Uchida et al. for example described a case series of 10 patients with SRSE and initiation of STP. The mean time to SE cessation was 30.8 days (range, 18–46 days) [15].

Based on previous data, STP may prove useful in the treatment of (super-)refractory SE.

## Methods

We retrospectively analyzed all patients who were admitted to the Department of Neurology, University Hospital Marburg, Germany, between 2013 and 2023 with a diagnosis of status epilepticus, who received additional treatment of SE with STP. We were able to identify 25 patients who received STP as add on therapy. All these patients suffered from RSE or SRSE. Since there was no SOP or treatment algorithm available, we have listed the individual treatment regimens in Table 2. 5 of these patients were previously published in a case series [13].

Treatment success was defined as continuous interruption of the SE.

Descriptive statistics are reported as absolute numbers and percentages, mean ± standard deviation (SD) or median ± median absolute deviation.

The study was approved by the local internal review board (IRB).

## Results

### Patients’ characteristics

A total of 25 patients (13 female and 12 male) were identified. The mean age was 58.6 ± 21.9 years (mean ± SD). All patients suffered from an at least refractory SE due to various aetiologies such as brain atrophy and subcortical arteriosclerotic encephalopathy (n = 3), subarachnoid hemorrhage in cerebral aneurysm (n = 2), post-meningioma resection, post-hemorrhagic defects, atypical Lennox-Gastaut syndrome, vascular lesions, post-infectious or unknown aetiology. The median dose was 1000 mg. STP was given as a compassionate use after several approved drugs for the treatment of SE, anesthetics, and other anti-seizure medication, such as levetiracetam, valproic acid, phenytoin, lacosamide, perampanel, zonisamide and brivaracetam; the median number of ASM in combination with STP was 4. The median number of previously given ASM was 6.

**Fig. 1:**
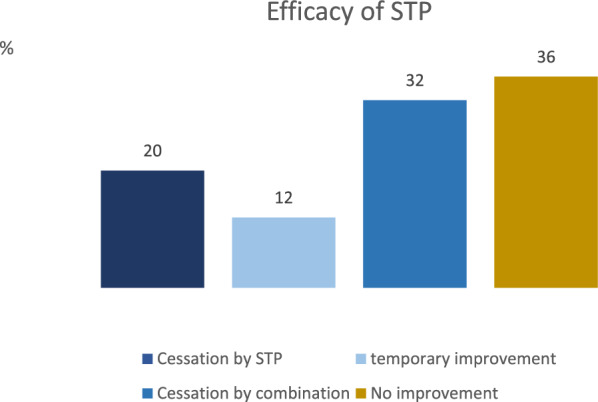
Efficacy of STP

Table 1 presents the characteristics of the patients, including demographic and clinical data. Treatment success was recorded in 64% of patients. In 20% of patients, the SE ceased after the additional administration of STP alone, and in 32% after the simultaneous administration of several ASMs, in particular VPA and benzodiazepines (Fig. 1). In 12% of patients, there was at least a temporary improvement in findings in the EEG or clinical. This improvement was also achieved by combining the ASM. Stiripentol only had to be discontinued in 3 patients due to a reduction in vigilance (2 patients) or hypercalcemia (1 patient).

**Table 1:** Participants’ demographic and clinical data

Patients’ characteristics	M (SD) %/[range]
**Demographic data**	n = 25
Age (years)	58.6 (21.9) [21–87]
*Sex*	
Female	52% (n = 13)
Male	48% (n = 12)
**Clinical data**	
**Aetiology**	
*Structural*	**72% (n = 18)**
Multiple older cerebral scars and hyperintense lesions	4% (n = 1)
Focal cortical dysplasia	4% (n = 1)
Early childhood brain damage	4% (n = 1)
Suspected Dyke-Davidoff-Masson syndrome	4% (n = 1)
Tuberous sclerosis	4% (n = 1)
Left hemimegalencephaly	4% (n = 1)
Amygdala hippocampectomy left with 2/3 temporal lobe resection	4% (n = 1)
Post-stroke	8% (n = 2)
Post-surgical	8% (n = 2)
Brain atrophy and subcortical arteriosclerotic encephalopathy	12 (n = 3)
Subarachnoid or intracerebral hemorrhage	16% (n = 4)
*Genetic*	4% (n = 1)
Progressive myoclonus epilepsy of the Lafora type 1	
*Infectious*	4% (n = 1)
Highly suspected sporadic Creutzfeldt-Jakob disease (CJD)	
*Unknown*	20% (n = 5)
Focal epilepsy without structural lesion	
**Treatment in combination with**	
- Benzodiazepines	100% (n = 25)
- Lorazepam	80% (n = 20)
- Clonazepam	4% (n = 1)
- Clobazam	12% (n = 3)
- Midazolam	4% (n = 1)
- Valproic acid	68% (n = 17)
- Levetiracetam	64% (n = 16)
- Zonisamide	4% (n = 1)
- Perampanel	20% (n = 5)
- Lacosamide	60% (n = 15)
- Topiramate	36% (n = 9)
- Brivaracetam	24% (n = 6)
- Lamotrigine	12% (n = 3)
- Phenytoin	8% (n = 2)
- Phenobarbital	4% (n = 1)
- Anesthesia	
- Propofol	16% (n = 4)
- Esketamine	12% (n = 3)
**Cessation of SE**	
- Cessation after STP alone	20% (n = 5)
- Improvement of results	12% (n = 3)
- Cessation after combination with other ASMs	32% (n = 8)
- No improvement	36% (n = 9)
**Discontinued if side effects occured (reduction in vigilance, hypercalcemia)**	12% (n = 3)

*M*: Mean, *SD*: Standard deviation

Since the order in which the anti-seizure medication is administered also influences the effect, the individual treatment regimes are shown in Table 2. If the patients had already taken anti-seizure medication beforehand, this is shown in brackets. In some cases, medications were already listed in the previous medication, but were given again to treat the SE, so that they are mentioned twice in these cases. It can be seen that the treatment regimes, analogous to the recommendations of the guideline, are very uniform in the first two stages of status therapy, but the subsequent sequence and administration of the various medications vary greatly. It should be noted here that in some patients the written or presumed will of the patient spoke against intubation. In addition, two patients had a primary purely focal status epilepticus, so that anaesthesia was delayed.

**Table 2:** Individualized order of the anti-seizure medication administered

Patient	Anti-seizure medication
1	BZD → LEV → LCM → VPA → STP
2	(ZNS, PER, LEV, VPA, CZP) → LZP → LEV → VPA → MDZ/ Esketamine → Cortisone → THP → CZP → LCM → PER → ZNS → STP
3	(LTG, LEV, VPA) → LZP → LEV → VPA → Propofol → MDZ → LCM → STP
4	(LEV) → LZP → LEV → LCM → VPA → BRV → STP
5	(LEV, ZNS, LCM) → LZP → LEV → VPA → LCM → Propofol → ZNS → MDZ → THP → TPM → PER → Cortisone → Esketamine → STP
6	(LEV) → LZP → LEV → VPA → LCM → STP
7	(LTG) → LZP → LEV → LCM → TPM → STP
8	(CLB, LCM, GBP, TPM) → LEV → STP
9	(LEV) → LZP → VPA → LCM → Propofol → PER → CLB → STP
10	(LEV, VPA, ESL, PER) → CLB → BRV → STP
11	BZD → LEV → VPA → LZP → LCM → TPM → STP
12	(LCM, LEV, VPA) → LZP → TPM → PER → Propofol → ESL → Cortisone → THP → Isoflurane → PHT → STP
13	(LEV, TPM, LCM, CLB) → LZP → MDZ/ Esketamine → Cortison → PB → TPM → STP
14	(LTG, PB, PER, BRV) → LZP → LEV → VPA → Propofol → MDZ/ Esketamine → TPM → THP → Cortisone → STP
15	(VPA, LEV, PER) → LZP → STP
16	(LEV, LTG) → LZP → LEV → LCM → PER → TPM → CBZ → Propofol → VPA → MDZ/ Esketamine → STP
17	BZD → LEV → VPA → Propofol → LCM → LZP → THP → TPM → STP
18	(VPA, LEV) → LZP → LEV → VPA → PER → LCM → STP
19	(VPA) → LZP → VPA → LEV → STP
20	(LEV) → LZP → LEV → LCM → TPM → STP
21	LZP → LEV → LCM → PER → STP
22	(LEV, VPA, LCM, PHT) → BRV → VPA → MDZ/ Esketamine → CLB → STP
23	(BRV, LCM) → LZP → VPA → Propofol → MDZ/ Esketamine → Cortisone → STP
24	LZP → LEV → LCM → Cortisone → TPM → BRV → Propofol → MDZ → VPA → STP
25	MDZ → LEV → LCM → BRV → VPA → STP

*BZD*: Benzodiazepine, *BRV*: Brivaracetam, *CLB*: Clobazam, *CZP*: Clonazepam, *LCM*: Lacosamide, *LEV*: Levetiracetam, *LTG*: Lamotrigine, *LZP*: Lorazepam, *MDZ*: Midazolam, *PB*: Phenobarbital, *PER*: Perampanel, *PHT*: Phenytoin, *STP*: Stiripentol, *TPM*: Topiramate, *VPA*: Valproic acid, *ZNS*: Zonisamide

The dose of medication administered also plays a major role. The exact dose of benzodiazepines could no longer be determined for all patients, but a dosage in line with the guidelines was generally selected. An exception here is the administration of benzodiazepines at first dose, which were often underdosed. In the majority of patients, only 1–2 mg lorazepam was administered; only 2 patients received 6 mg fractionally.

Of the 25 patients mentioned, seven died during hospitalisation as a result of complications caused by the SE or a treatment limitation in accordance with the patient’s written or presumed wishes. Survival of more than 5 years was observed in nine patients, three patients had a 2-year survival. One patient was discharged to a palliative setting, while the course of five others remained unclear after transfer to rehabilitation.

## Discussion

The study reports on 25 adult patients with (super-)refractory status epilepticus (SE) who had been treated with STP as an additional antiseizure medication (ASM). The study reports that in 64% of the 25 patients (13 female) SE ceased after the administration of STP. In 32% of the patients, SE ceased after STP therapy alone. In the remaining 32%, SE ceased after the administration of STP in combination with other ASM. The use of STP was well tolerated in these critically ill patients. Despite the limitations of a small cohort, the data suggests that STP may be a novel therapeutic option for the treatment of patients with RSE and SRSE. Super-refractory status epilepticus is resistant to benzodiazepines, anti-seizure medication, and general anesthesia. Pharmacoresistance to benzodiazepines develops rapidly after SE onset and is due to an activity-dependent internalization of benzodiazepine-sensitive synaptic GABA_A_ (c-aminobutyric acid) receptors during SE [5]. STP is a positive allosteric modulator of GABA_A_ receptors. It enhances GABA_A_-mediated inhibition, including potentiation of miniature inhibitory postsynaptic currents by slowing the decay rate [1, 7]. These results of an experimental study suggest that, at doses that result in therapeutically relevant concentrations, STP may mediate its anticonvulsant effects by potentiating GABAergic inhibition. The subunit selectivity profile of STP allows it to remain effective despite GABA_A_ receptor subunit changes in the course of SE. The additive effects of benzodiazepines (BZDs) and STP observed suggest that the combination of STP and benzodiazepines would produce a greater enhancement of GABAergic inhibition and a greater variety of GABA_A_ receptors than would BZDs alone. These findings point to the potential use of STP, either alone or as add-on therapy, for treatment of established and BZD-resistant SE [7].

Several case reports and small series suggest that STP might be a treatment option for refractory SE: In 2018, Uchida et al. identified 10 adult patients with super-refractory status epilepticus (SRSE) to investigate the optimal ASM for the treatment of SRSE in patients with cross-sensitivity. Stiripentol was administered in five patients that had shown cross-sensitivity when other ASMs were used and failed to control seizures [15]. To determine whether STP could be a treatment option for SRSE, [13] reviewed medical records of patients with refractory SE that had been treated with STP between January 2013 and June 2014. Primary endpoints were the termination of SE after the start of STP. Five adult patients were started on STP due to SRSE. The median age was 78 years (interquartile range [IQR] 11 years), and four patients were female. The median duration of SRSE before initiation of STP was 39 days (IQR 16 days) and the median number of anticonvulsants previously used was 6 (IQR 1). SRSE ceased in three patients within 2–4 days after the start of STP. In two patients, SRSE continued after administration of STP and further escalation of anticonvulsant treatment. Both were eventually switched to supportive care only. No serious side effects were observed during treatment with STP [13]. The present study can be seen as an extension or continuation of the 5 documented patients. It was thus possible to confirm in a larger cohort that side effects play practically no role in this context. In our cohort, the median number of previously given ASM was 6 and thus also comparable with the case series from 2015.

In our 2021 study, in which 22 patients with refractory epilepsy were examined with regard to the use of STP, it was shown that after 6 months 31% of patients were responders, including 13% who were seizure-free [8]. In the present study, the success rates were slightly higher compared to 2021, which can most likely be attributed to the additional administration of benzodiazepines and valproate in particular.

Stiripentol had to be discontinued in 1 patient due to hypercalcemia 1 patient. The hypercalcemia cannot be explained pathophysiologically by the addition of STP. Since the calcium value did not normalize after discontinuation of the medication, a causal relationship cannot be assumed.

Furthermore, the mortality at discharge was 28%, which puts the present cohort within the range of the previously published mortality rates of the RSE [10].

To the best of our knowledge, the study presented here currently analyzes the largest study cohort to date with 25 patients and is therefore of particular relevance for assessing the effect of STP in the treatment of SRSE. In our cohort, the mean number of ASMs combined with STP was 4.5 (range 2–9) and therefore half of the mean number of ASMs in the described study. Nevertheless, an equally good efficacy could be demonstrated. In addition to the studies available to date, we were able to report an extremely positive effect with almost no documented side effects.

## Limitations

It should be noted that the retrospective study design and small group size represent the limitations of this study. Moreover, the cohort under examination is highly heterogeneous with regard to the etiology of SE, which is reflective of clinical practice. The assessment of treatment for refractory SE is inherently challenging, as this stage of treatment typically involves the simultaneous administration of multiple ASMs. Consequently, the assessment of the effect of a single ASM is often impractical, as a combination of ASMs is frequently the most effective approach.

## Conclusions

STP may represent a promising additional treatment option for refractory and super-refractory status epilepticus. Tolerability has already been demonstrated in previous studies, which is also reflected in these data. The development of efficacious treatments for refractory SE is a pressing necessity. It would be beneficial to conduct prospective studies in to more accurately prove the benefit of STP in the treatment of SE.

## Data Availability

The datasets used and analysed during the current study are available from the corresponding author on reasonable request.

## Abbreviations

ASM: Anti-seizure medication
BRV: Brivaracetam
BTCS: Bilateral tonic–clonic seizures
BZD: Benzodiazepine
CLB: Clobazam
CZP: Clonazepam
DS: Dravet syndrome
EEG: Electroencephalography
GABA A: C-aminobutyric acid
ICU: Intensive care unit
IRB: Institutional Review Board
LCM: Lacosamide
LEV: Levetiracetam
LTG: Lamotrigine
LZP: Lorazepam
M: Mean
MDZ: Midazolam
PB: Phenobarbital
PER: Perampanel
PHT: Phenytoin
RSE: Refractory status epilepticus
SD: Standard deviation
SE: Status epilepticus
SRSE: Super-refractory status epilepticus
STP: Stiripentol
TPM: Topiramate
VPA: Valproic acid
ZNS: Zonisamide
